# Ethanol Extract of *Centella asiatica* (Gotu Kola) Attenuates Tubular Injury Through Inhibition of Inflammatory Cytokines and Enhancement of Anti-Fibrotic Factor in Mice with 5/6 Subtotal Nephrectomy

**DOI:** 10.21315/mjms2019.26.5.5

**Published:** 2019-11-04

**Authors:** Nur Arfian, Wiwit Ananda Wahyu Setyaningsih, Nungki Anggorowati, Muhammad Mansyur Romi, Dwi Cahyani Ratna Sari

**Affiliations:** 1 Department of Anatomy, Faculty of Medicine, Public Health and Nursing, Universitas Gadjah Mada, Yogyakarta, Indonesia; 2 Department of Anatomical Pathology, Faculty of Medicine, Public Health and Nursing, Universitas Gadjah Mada, Yogyakarta, Indonesia

**Keywords:** 5/6 subtotal nephrectomy, Centella asiatica, tubular injury, anti-inflammatory, anti-fibrotic

## Abstract

**Background:**

Chronic kidney disease (CKD) leads to inflammation, fibrosis and destruction of the renal architecture. *Centella asiatica* (CeA) is an herbaceous plant with anti-inflammatory effects. We aimed to elucidate the effect of CeA on inflammation, fibrosis, vascular remodelling and antifibrotic substances in a 5/6 subtotal nephrectomy (SN) model in mice.

**Methods:**

Mice were divided into three groups: sham operation (SO, *n* = 6), 5/6 SN for seven days (SN7, *n* = 7) and SN7 with oral CeA treatment (SN7-CeA, *n* = 7). At day 7, mice were euthanised, kidneys were harvested and stained with periodic-acid Schiff (for tubular injury and glomerulosclerosis) and sirius red (for fibrosis and vascular remodeling) staining. mRNA expression of prepro-endothelin-1, nephrin, E-cadherin, bone morphogenic protein-7 (BMP-7), toll-like receptor 4 (TLR4), tumour necrosis factor-α (TNFα) and hepatocyte growth factor (HGF) were quantified using reverse transcriptase-PCR.

**Results:**

SN group demonstrated significant higher interstitial fibrosis, vascular remodeling, tubular injury and glomerulosclerosis (*P* < 0.01) compared to SO group. Meanwhile, in SN7-CeA demonstrated attenuation of vascular remodeling as shown by significant higher lumen area with lower Wall/Lumen area ratio compared to SN7. RT-PCR analysis showed up-regulation of nephrin, BMP-7 and E-cadherin mRNA expression (*P* < 0.05) and down-regulation of ppET-1 in SN7-CeA group compared to SN7 group (*P* < 0.05).

**Conclusion:**

*CeA* may ameliorate renal injury in the SN model in mice.

## Introduction

Chronic kidney diseases (CKDs) lead to kidney fibrosis and destruction of the renal architecture. CKDs also induce renal inflammation, glomerulosclerosis and interstitial fibrosis. These conditions contribute to increased morbidity and mortality rates. Several factors that contribute to the development of chronic kidney disease include hypertension, diabetes and dyslipidaemia. Subtotal nephrectomy (SN), also called 5/6 nephrectomy, is an animal experimental model of CKD. It is performed by uninephrectomy and followed by 2/3 ablation of the contra-lateral kidney. The reduction in the glomerular filtrate rate (GFR) is prominent after two weeks of treatment. Arterial hypertension, known as the antecedent of the decline in renal function, results in glomerulosclerosis and tubulointerstitial injury (1–3).

Glomerulosclerosis manifests as defects of the glomerular filtration barrier. Podocytes become injured due to damage of slit-diaphragm (SD). Nephrin and podocin are the components of the podocyte and slit-diaphragm which are encoded by the NPHS1 and NPHS2 genes, respectively. Renal injury breaks the actin cytoskeleton, causing foot-process effacement, detachment of podocytes from the glomerular basement membrane and urinary protein loss (4, 5). Most kidney disease is glomerular in origin, while tubulointerstitial fibrosis is the strongest indicator of disease progression. Prolonged and persistent injury is characterised by excessive proliferation of the extracellular matrix and results in fibrosis or sclerosis (6, 7).

Many pathways play roles in the mechanisms of CKDs and kidney fibrosis, such as the endothelin-1 (ET-1) and transforming growth factor-beta 1 (TGF-β1) pathways. ET-1 is a potent vasoconstrictor observed for the first time by Yanagisawa (8); furthermore upregulation of ET-1 in ET-1 transgenic mice leads to spontaneous kidney fibrosis (9). Furthermore, TGF-β1 is a factor key of kidney fibrotic which plays important roles in myofibroblast activation and interstitial matrix deposition (10). Antifibrotic factors are produced to respond to the action of TGF-β1 during fibrogenesis. Several studies have documented hepatocyte growth factor (HGF) and bone morphogenic protein-7 (BMP-7) as kidney anti-fibrotic factors (11, 12). HGF counteracts the action of TGF-β1 by inhibiting TGF-β1-mediated myofibroblast activation and blocking extracellular matrix deposition (13). BMP-7 is a member of the TGF-β superfamily that promotes anti-fibrotic action by blocking the TGF-β1/Smad3 pathway (12, 14).

*Centella asiatica* (CeA) is an herbaceous plant that is well-recognised to decrease fibrosis in the skin, lungs and liver (15, 16). Some mechanisms of *CeA* action in fibrosis have been investigated (16–18). Asiatic acid is one of the bioactive components of *CeA* that is known to decrease fibrosis through Smad7, which is an inhibitory Smad of the TGF-β1/Smad3 pathway (7, 17). Through inhibition of the TGF-β/Smad, asiatic acid could prevent myofibroblast activation and the matrix deposition. *CeA* also demonstrates anti-inflammatory activity by the inhibition of inflammatory factors, such as IL-6, IL-1β, and TNF-α (16, 19). Nevertheless, the effect of *CeA* on kidney anti-fibrotic factors, such as HGF and BMP-7, remains largely unknown. Therefore, we aimed to elucidate the effect of *CeA* on kidney interstitial fibrosis, vascular remodelling, and upregulation of antifibrotic substances, such as HGF and BMP-7, in a 5/6 SN model.

## Materials and Methods

### Animal Experiment and the 5/6 SN Model

This study was performed with approval from the Ethical Committee of Faculty of Medicine, Public Health and Nursing of Universitas Gadjah Mada based on a statement letter of ethical expedience (KE/FK/0405/EC/2017, 20 April 2017). Male Swiss background mice (*n* = 27, 3 months, 30 g–40 g) were obtained from the Experimental Animal Care Unit of Universitas Gadjah Mada, Yogyakarta, Indonesia. The mice were divided into three groups randomly: a sham operation (SO) group, an SN7 group (using the 5/6 SN procedure) and an SN7-*CeA* group (5/6 SN with *CeA* 840 mg/kg treatment orally) (20). The 5/6 SN was performed to induce CKD and kidney fibrosis. Mice were anesthetised with sodium pentobarbital (10 mg/kg) intraperitoneally. The abdomen was opened in the right flank region, and the right kidney was removed (unilateral nephrectomy). Polar excision of the left kidney was performed on the second day (SN). Mice were housed in a 50 cm × 30 cm × 15 cm plastic cage according to their group with a maximum of three mice in each cage. The cage environment was kept at a 12:12-h natural light-dark cycle, 21 °C temperature and humidity of 40%–60%. The mice were fed with standard food and water *ad libitum*.

### Kidney Harvesting

Mice were euthanised at day 7 after the operation. Mice were anesthetised with sodium pentobarbital (10 mg/kg) intraperitoneally and then the abdomen and thorax were opened to visualise the heart and kidney. The organs were perfused with 0.9% NaCl from the left ventricle using a Perista pump (Atto^®^; Cat. No. SJ-1211H). The left kidney was harvested, with one half kept in RNA later^®^ for RNA extraction and the other half was fixed in 4% PFA in PBS for paraffinisation.

### Fibrosis Area Fraction, Glomerulosclerosis and Tubular Quantification

Paraffin sections of 4 μm thickness were deparaffinised and stained with Sirius red (SR) to quantify the fraction area of interstitial fibrosis. The image was captured using the OptiLab software (Olympus; Cat. No. CX22) at 400× magnification of 15 fields for each sample. The areas were randomly chosen in the cortex and medulla. The interstitial fibrosis area fraction was quantified using ImageJ software.

The extent of glomerulosclerosis (GS) was scored based on periodic acid Schiff’s (PAS) staining using the following criteria: extent of glomerular damage and matrix expansion (sclerosis), capillary loops and synechia between glomerular capillaries and the Bowman’s capsule. The glomerulus was graded as follows: 0 = normal; 1 = mesangial expansion/sclerosis involving < 25% of the tuft; 2 = moderate GS (25% to 50%); 3 = severe GS (50% to 75%); and 4 = diffuse GS (involving > 75% of the glomerular tuft). For each kidney, the sum of the results for 20 glomeruli was defined as the glomerulosclerosis index (GSI). The GSI of each mouse was calculated as a mean value of all the glomerular scores obtained.

The tubular injury score was assessed based on the histopathology of the tubules. Ten to fifteen fields with 400× magnification were examined for each kidney, and the lesions were graded from 0 to 3 (0 = no change; 1 = changes affecting < 25% of the section; 2 = changes affecting 25% to 50% of the section; and 3 = changes affecting 50% to 100% of the section), according to the area with tubulointerstitial lesions (tubular atrophy, tubular dilatation, loss of brush-border intraluminal casts, interstitial inflammation and fibrosis). The score index of each mouse was expressed as a mean value of all scores obtained.

### RNA Extraction, cDNA Synthesis and RT-PCR

The RNA from kidney tissue was extracted using Trizol RNA solution (GENEzol^TM^; Cat. No. GZR100) and the RNA concentration was quantified using the Nanodrop system. The cDNA was synthesised using ReverTra-Ace (Toyobo^®^; TRT-101) with the addition of random primers (TAKARA^®^, 3801) and dNTP (TAKARA^®^, 4030). Reverse transcription-polymerase chain reaction (RT-PCR) was performed to examine the following genes:

**Table:** 

No.	Gene	Sequences	Annealing temperature
1	Nephrin	F: 5′-CCCCTCTATGATGAAGTACAAATGGA-3′	57 °C
		R: 5′-GTACGATTTCCTCAGGTCTTCT-3′	
2	E-Cadherin	F: 5′-CAGCCTTCTTTTCGGAAGACT-3′	58 °C
		R: 3′-CAGCAAGAAGAGGTCCGACT-3′	
3	BMP-7	F: 5′-CGAGACCTTCCAGATCACAGT-3′	57 °C
		R: 5′-CAGCAAGAAGAGGTCCGACT-3′	
4	HGF	F: 5′-CATTCAAGGCCAAGGAGAAG-3′	54 °C
		R: 5′-AACTGGATGTTTGGGTCAG-3′	
5	TNFα	F: 5′-AGGCACTCCCCCAAAAGATG-3′	60 °C
		R: 5′-CCACTTGGTGGTTTGTGAGTG-3′	
6	TLR4	F: 5′-GGGCCTAAACCCAGTCTGTTTG-3′	57°C
		R: 5′-GCCCGGTAAGGTCCATGCTA-3′	
7	NFκB	F: 5′-GCGTACACATTCTGGGGAGT-3′	57 °C
		R: 5′-ACCGAAGCAGGAGCTATCAA-3′	
8	GAPDH	F: 5′-TGTGTCCGTCGTGGATCTGA-3′	57 °C
		R: 5′-TTGCTGTTGAAGTCGCAGGAG-3′	

The reagent amounts were based on the kit instructions (Go Taq master Mix; M7122). The PCR conditions were as follows: 94 °C denaturation for 10 s, annealing at 60 °C for 30 sec, extension 72 °C for 1 min and a final extension phase at 72 °C for 10 min.

### Statistical Analysis

Normally distributed data were analysed using one-way ANOVA and the Kruskal-Wallis test was used for data that were not normally distributed. A value of *P* < 0.05 indicated statistical significance. Statistical analyses were performed using SPSS software version 22.0 (SPSS Inc., Chicago).

## Results

### CeA Attenuates Glomerulosclerosis, Tubular Injury and Interstitial Fibrosis

**Figure 1:**
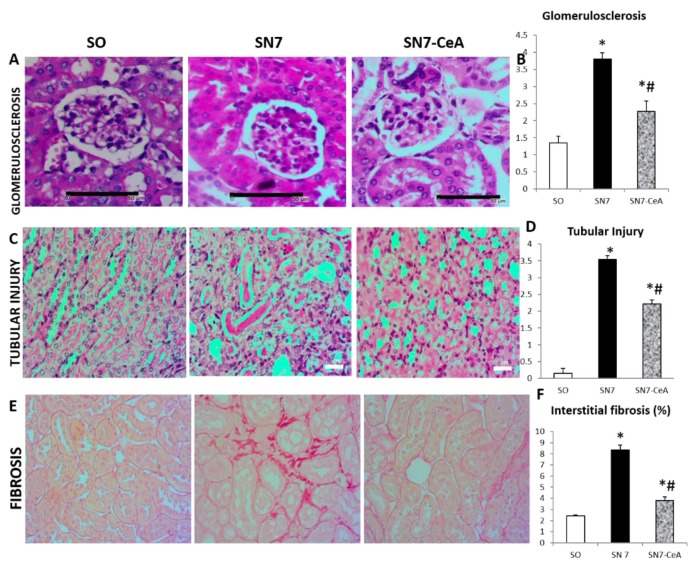
**A–B**: Representative images of glomerulosclerosis and quantification of the glomerulosclerosis score. The SN group demonstrated glomerulosclerosis with deposition of the extracellular matrix in the glomerulus and capillary tuft closing. **C–D**: Tubular injury quantification based on tubular injury (dilatation, intraluminal cast formation, brush border loss and epithelial cell effacement). **E–F**: Sirius red staining showed interstitial fibrosis with red-coloured staining in the kidney after SN. *P < 0.05 versus SO, #*P* < 0.05 versus SN7

The 5/6 SN model represents chronic renal failure with deteriorating kidney function. Glomerulosclerosis found in the SN7 group was characterised by accumulation of the extracellular matrix, capillary tuft closing and synechiae in the glomerulus (Figure 1A). Glomerulosclerosis was followed by tubular injury in PAS staining with tubular dilation, intraluminal cast deposition, effacement of epithelial cells and brush border loss (Figure 1B). The final renal architecture damage event, interstitial fibrosis, was also found in the SN7 group based on the red colour of Sirius red staining. The glomerulosclerosis score (*P* = 0.000), tubular injury score (*P* = 0.009), and interstitial fibrosis area fraction (*P* = 0.000) were significantly higher in the SN7 group than in the SO group. On the other hand, the SN7-*CeA* group demonstrated a significantly lower glomerulosclerosis score (*P* = 0.000), tubular injury score (*P* = 0.014) and interstitial fibrosis area fraction (*P* = 0.000) quantification than the SN7 group. There were significant differences between the SN7-*CeA* and SO groups in the glomerulosclerosis score (*P* = 0.000), tubular injury score (*P* = 0.014), and interstitial fibrosis area fraction (*P* = 0.002).

### CeA Attenuates Vascular Remodelling by Downregulating E-cadherin mRNA Expression

We investigated podocyte injury and epithelial injury in this model. SN also induced detachment of podocytes and epithelial injury, as shown by lower nephrin (podocyte marker) and E-cadherin (epithelial cell marker) mRNA expression in the SN group compared to the SO group. Additionally, the SN7-*CeA* group demonstrated higher nephrin (*P* = 0.034) and E-cadherin (*P* = 0.025) expression than the SN7 group. Lumen area quantification was performed to demonstrate vascular remodelling. The SN group had a smaller lumen area than the SO group, a finding that was associated with a higher wall/lumen area ratio (WLAR). On the other hand, the SN7-*CeA* group demonstrated a significantly greater lumen area (*P* = 0.002) with a lower WLAR (*P* = 0.020) than the SN7 group. These results confirmed the attenuation of vascular remodelling in the SN7-*CeA* group.

### CeA Inhibits Inflammatory Cytokines and Enhances Anti-Fibrotic mRNA Expression

**Figure 2:**
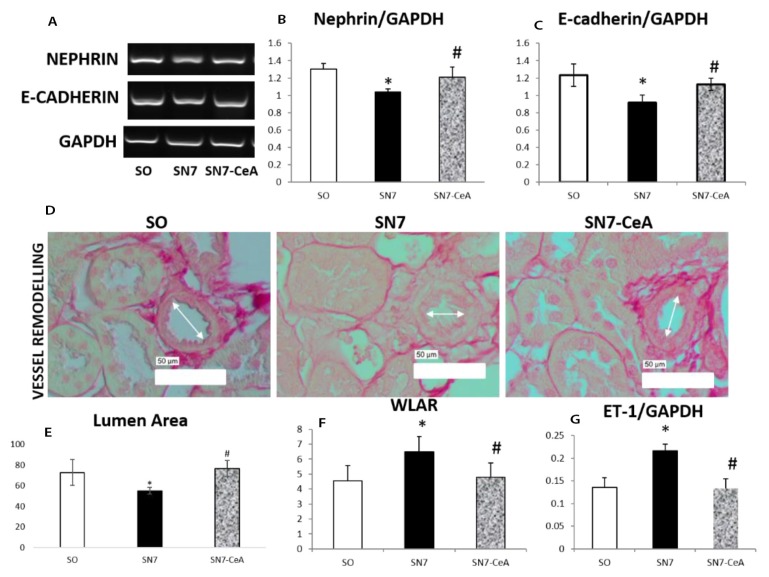
**A–C**: Representative images of nephrin and E-cadherin mRNA expression based on RTPCR quantification. **D–F**: The lumen area and wall/lumen area ratio quantification are represent vascular remodelling. **G**: ET-1 mRNA quantification based on RT-PCR analysis. Bar = 50 μm. *P < 0.05 versus SO, #*P* < 0.05 versus SN

**Figure 3:**
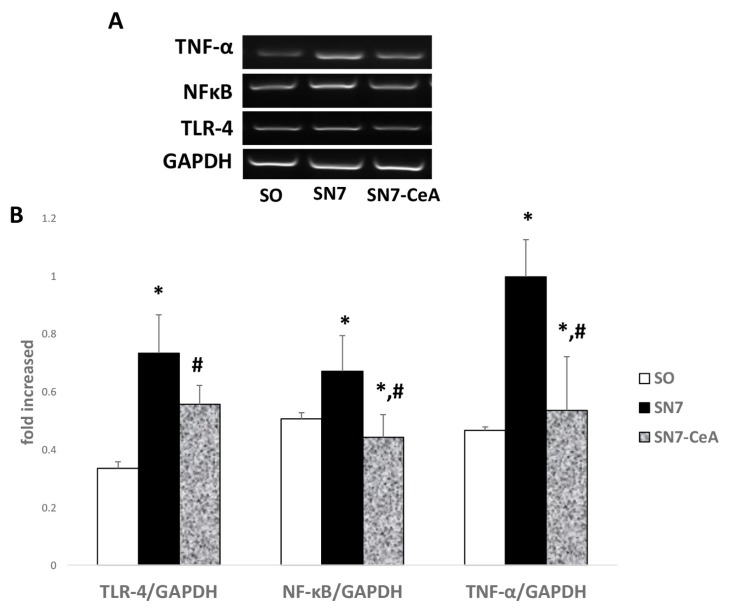
*CeA* inhibits inflammatory cytokines in CKD. **A**: Representative images of inflammatory cytokines expression using RT-PCR. **B**: Densitometry analysis of reverse transcription-PCR showed reduction of inflammatory cytokines in the SN-CeA group. *P < 0.05 versus SO, #*P* < 0.05 versus SN

Inflammation contributes to the progression of CKD through the release of inflammatory cytokines and increases both the production and activity of the adhesion molecules. Here, we showed that 5/6 SN group as a CKD model showed higher mRNA expression of inflammatory cytokines, such as TNFα (*P* = 0.007) and TLR4 (*P* = 0.000), followed by increases in NFκB (*P* = 0.041) than the SO group. Additionally, after *CeA* (SN7-*CeA*) treatment, the NFκB (*P* = 0.009), TNFα (*P* = 0.012), and TLR4 (*P* = 0.033) mRNA expression levels of inflammatory cytokines were decreased compared with those of the SN group (Figure 3). ET-1 is a potent vasoconstrictor that has been known to play a role in kidney injury and fibrosis. The mRNA quantification revealed upregulation of ppET-1 mRNA in the SN7 group (*P* = 0.013). This might be associated with a lower lumen area and higher WLAR representing vascular remodelling in the SN7 group (Figure 2).

RT-PCR also revealed significantly lower BMP-7 (*P* = 0.034) and HGF (*P* = 0.004) mRNA expression in the SN7 group than that in the SO group. Additionally, the SN7-CeA group showed significantly higher BMP-7 expression (*P* < 0.05) than the SN7 group and the expression was statistically the same as that of the SO group, but not for HGF.

## Discussion

Here, we reported the improvement of kidney injury after *CeA* treatment in 5/6 SN by reducing inflammation, vascular remodelling and fibrosis. SN induces renal disease progressivity, which represents CKDs. The early phases of SN are characterised by glomerulosclerosis with early tubular injury (1, 2). These injuries were characterised by deposition of the extracellular matrix, resulting in sclerosis or fibrosis. After treatment with *CeA*, the glomerulosclerosis and tubulointerstitial injury scores decreased (Figure 1). These findings are associated with amelioration of the mRNA expression of nephrin as a podocyte marker and E-cadherin as an epithelial cell marker. The slit diaphragm protein nephrin is a transmembrane protein that is found between podocyte foot processes. Mutation of the nephrin causes massive proteinuria. Podocin, like nephrin, is present in the podocyte plasma membrane in the area of the slit diaphragm (21). Thus, decreased nephrin and podocin affects the structure and function of podocytes. *CeA* treatment in an adriamycin-induced nephropathy model showed increased podocin, nephrin, and synaptopodin mRNA and protein levels (18). The increase in podocytes after *CeA* treatment enhanced the histological changes (Figure 1). The renoprotective effects of CeA may also be due to the compounds in the CeA extract. *CeA* contains many chemical triterpenoid compounds, such as asiaticoside, madecassoside, madecassic acid and asiatic acid (22).

Vascular remodelling plays a significant role in the progression of kidney fibrosis and ischemia (23). Some molecules that regulate vessel tonus balance, such as ET-1 and NO, influence vessel remodelling (24). We revealed that vascular remodelling with a reduced lumen area and increased WLAR occur in SN (Figure 2D–F) and are associated with the upregulation of ppET-1 mRNA. Kidney fibrosis induces ischemia with an imbalance between vasoconstrictor and vasodilation factors (23). ET-1 is a peptide hormone that regulates many physiological functions (25). Various conditions, such as hypoxia and shear stress, are known to stimulate the upregulation of ET-1 through the exocytosis of Weibel-Palade bodies (26). ET-1 and ETAR activation occurred in intrarenal artery remodelling in a kidney ischemia/reperfusion injury model. Deletion of ET-1 from EC ameliorates vascular remodelling with a reduction in the wall thickness and may be associated with ETAR reduction and attenuation of IR injury (27). The reduction of NO might induce vasoconstriction produced by endothelial damage (28). Vascular remodelling and kidney fibrosis have also been reported in mice with a deletion of the prolyl hydroxylase domain protein-2 from endothelial cells (29).

Kidney damage provokes interstitial inflammation and tubular activation. Inflammatory cells, such as lymphocytes, macrophages, dendritic cells, and mast cells, infiltrate into the interstitial space and become activated (30–32). Activated macrophage M1 releases inflammatory cytokines, such as TNFα, IL-1β and IL-6, while macrophage M2 releases TGF-β1 and IL-10 to initiate inflammatory resolution (33). We found that the 5/6 SN mouse model upregulates NFκB, TNFα and TLR4, and was impeded after *CeA* treatment for seven days. *CeA* is known for its anti-inflammatory activity due to its ability to downregulate inducible nitric oxide synthase (iNOS), interleukin (IL-1β, IL-6) and cytokines such as TNF-α and cycloxygenase2 (COX2) through the inhibition of NFκB activation (16, 19, 34). Under the unstimulated condition, NFκB will be sequestered in the cytosol by its inhibitor IκB. Under stimulation of the lipopolysaccharide (LPS), IκB undergoes phosphorylation. Yun et al. showed that *CeA* reduces LPS-induced p-IκB-α/β phosphorylation in a dose-dependent manner (35).

Inflammatory cytokines, growth factors, and prostaglandin that are produced during kidney damage regulate the expression of HGF (36). Along with the progression of fibrosis, HGF expression was documented to be lower than that in the control group, similar to BMP-7 mRNA expression. *CeA* treatment impedes either HGF or BMP-7 mRNA expression (Figure 3). Some studies have suggested that HGF expression is regulated by inflammatory cytokines, growth factors and prostaglandin (36). Induction of HGF expression is high in organs distant from the injured area, while HGF receptor (c-Met) expression is selectively high in the fibrotic area (37, 38). Circulatory hormone, growth factors, and cytokines may influence the expression and distribution of HGF from distant organs to fibrotic organs (39). *CeA* treatment enhances anti-fibrotic mRNA expression of both BMP-7 and HGF in CKD. This study confirmed findings in the research conducted by Zhang et al. (6) that showed high doses of *CeA* increased both the mRNA and protein levels of HGF in a unilateral ureteral obstruction (UUO) mouse model. The alteration of anti-fibrotic factor was followed by rectification of inflammatory cytokines, such as MCP-1 (6). The anti-fibrotic effect of asiatic acid inhibits Smad3, resulting in increases of both Smad7 protein and mRNA expression. Inhibiting the TGF-β/Smad downstream signalling pathway prevents myofibroblast activation and matrix deposition. Inhibition of the TGF-β/Smad signalling pathway also reduces the production of TGF-β-mediated profibrotic factors such as connective tissue growth factor (CTGF), alpha-smooth muscle actin (α-SMA), collagen I and plasminogen activator inhibitor-1 (PAI-1) (7, 40).

## Conclusion

*CeA* treatment impedes renal injury after seven days in a 5/6 SN model by inhibiting inflammatory cytokines and enhancing anti-fibrotic factors.

## Figures and Tables

**Figure 4:**
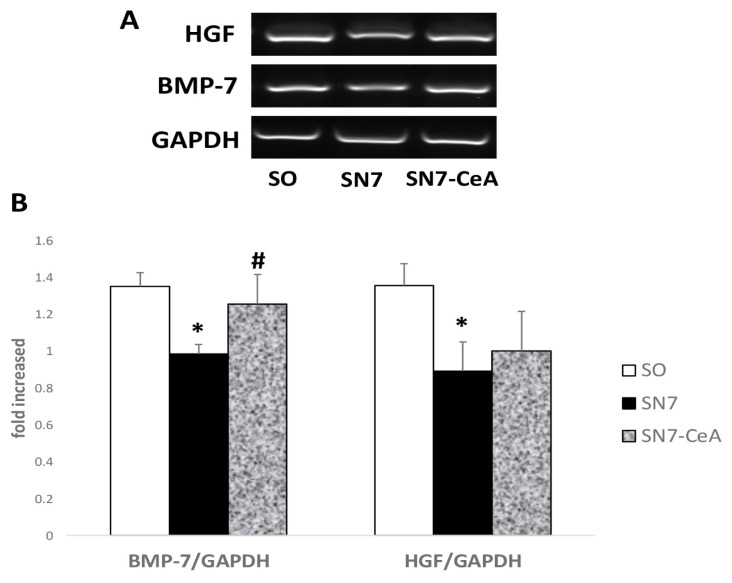
*CeA* impedes anti-fibrotic factors. **A**: Representative images of anti-fibrotic ligand expression using RT-PCR. **B**: Densitometry analysis of reverse transcription-PCR showed alteration of the anti-fibrotic effect in the SN-CeA group. *P < 0.05 versus SO, #*P* < 0.05 versus SN
